# Examination of mid-intervention mediating effects on objectively assessed sedentary time among children in the Transform-Us! cluster-randomized controlled trial

**DOI:** 10.1186/1479-5868-10-62

**Published:** 2013-05-20

**Authors:** Valerie Carson, Jo Salmon, Lauren Arundell, Nicola D Ridgers, Ester Cerin, Helen Brown, Kylie D Hesketh, Kylie Ball, Mai Chinapaw, Mine Yildirim, Robin M Daly, David W Dunstan, David Crawford

**Affiliations:** 1 Faculty of Physical Education and Recreation, University of Alberta, Edmonton, AB, Canada; 2 Centre for Physical Activity and Nutrition Research, School of Exercise and Nutrition Sciences, Deakin University, Melbourne, VIC, Australia; 3 Depertment of Public and Occupational Health, EMGO Institute for Health and Care Research, VU University Medical Center, Amsterdam, The Netherlands; 4 Baker IDI Heart and Diabetes Institute, Melbourne, VIC, Australia

**Keywords:** Sedentary behavior, Intervention, Mediators, Children

## Abstract

**Background:**

The optimal targets and strategies for effectively reducing sedentary behavior among young people are unknown. Intervention research that explores changes in mediated effects as well as in outcome behaviors is needed to help inform more effective interventions. Therefore, the purpose of this study was to examine the mid-intervention mediating effects on children’s objectively assessed classroom and total weekday sedentary time in the Transform-Us! intervention.

**Methods:**

The results are based on 293 children, aged 7- to 9-years-old at baseline, from 20 schools in Melbourne, Australia. Each school was randomly allocated to one of four groups, which targeted reducing sedentary time in the school and family settings (SB; n = 74), increasing or maintaining moderate- to vigorous-intensity physical activity in the school and family settings (PA; n = 75), combined SB and PA (SB + PA; n = 80), or the current practice control (C; n = 64). Baseline and mid-intervention data (5–9 months) were collected in 2010 and analyzed in 2012. Classroom and total weekday sedentary time was objectively assessed using ActiGraph accelerometers. The hypothesized mediators including, child enjoyment, parent and teacher outcome expectancies, and child perceived access to standing opportunities in the classroom environment, were assessed by questionnaire.

**Results:**

The SB + PA group spent 13.3 min/day less in weekday sedentary time at mid-intervention compared to the control group. At mid-intervention, children in the SB group had higher enjoyment of standing in class (0.9 units; 5-unit scale) and all intervention groups had more positive perceptions of access to standing opportunities in the classroom environment (0.3-0.4 units; 3-unit scale), compared to the control group. However, none of the hypothesized mediator variables had an effect on sedentary time; thus, no mediating effects were observed.

**Conclusions:**

While beneficial intervention effects were observed on some hypothesized mediating variables and total weekday sedentary time at mid-intervention, no significant mediating effects were found. Given the dearth of existing information, future intervention research is needed that explores mediated effects. More work is also needed on the development of reliable mediator measures that are sensitive to change overtime.

**Trial registration:**

ACTRN12609000715279

ISRCTN83725066

## Background

There is a growing body of evidence that excessive sedentary behavior (i.e., too much sitting), in particular screen-based sedentary behavior, is an important determinant of overweight/obesity and related cardio-metabolic biomarkers among children [1]. Further, some evidence suggests that these effects are independent of children’s moderate-to vigorous-intensity physical activity (MVPA) [2]. While screen-based sedentary behavior is common among young people, recent studies suggest that it is not a good proxy of the total sedentary time for a given day [3,4]. For instance, children may accumulate high amounts of sedentary time through being transported to school, during class time, and when doing homework. Evidence of associations between objectively assessed total sedentary time and children’s health is an emerging research area. While findings of existing studies have been inconsistent [2,5-9], it appears that levels of total sedentary time may track overtime [10]. Among adults, more consistent associations have been observed between total sedentary time and cardio-metabolic biomarkers [11-13]. Therefore, interventions that target the reduction of total sedentary time among children are of public health importance.

Since the potential importance of reducing children’s total sedentary time has only recently been identified, previous research has primarily targeted children’s screen-based sedentary behavior [14-16]. However, meta-analyses of the effectiveness of interventions to reduce children’s screen time have produced mixed results. For example, Wahi and colleagues did not find a significant decrease in screen time (−0.91 hr/week) among children under 18 years-old across nine randomized controlled trials [16]. In contrast, Kamath and colleagues reported a small but significant reduction (*Cohen d* = −0.29) in screen time among 2- to 18-year-olds across 14 randomized controlled trials [15]. Because the mediators or mechanisms of behavior change have not been examined in any of these earlier studies, it is difficult to determine why the interventions included in these reviews reported mixed effects on screen time. In fact, a recent systematic review on mediating mechanisms of school-based energy balance behavior interventions only identified three studies that examined potential mediators of screen time interventions [17]. Among these three studies, no significant mediators were identified [17].

Given that few intervention studies targeting screen time have conducted mediation analyses, it is difficult to draw meaningful conclusions regarding the optimal targets and strategies for effectively reducing screen time among young people. Less is known about effectively reducing total sedentary time, since no study has examined potential mediating effects. Further, most studies have relied on self- or proxy-report measures of sedentary behavior, which limit the ability to accurately determine the intervention effects [18]. Therefore, the purpose of this study was to expand the knowledge base in this area by examining the mid-intervention mediating effects on children’s objectively assessed classroom and total weekday sedentary time in the Transform-Us! study [19].

Transform-us! is an 18-month, four-arm cluster-randomized controlled trial within primary (elementary) schools in Melbourne, Australia aiming to increase children’s physical activity, decrease sedentary behavior, and optimize health outcomes. The study is based on elements of the social cognitive theory [20], behavioral choice theory [21], and the ecological systems theory [22]. Assessing whether changes have occurred in targeted mediators as well as in outcome behaviors at mid-intervention can help determine whether the study is proceeding as planned. This knowledge can help in the understanding and the evaluation of future intervention and mediation effects.

## Methods

### Participants

Children recruited from schools within a 50 km radius of Melbourne Central Business District that had an enrolment greater than 300, at least two Grade 3 classes, co-educational, and located in the first (low; n = 74), third (mid; n = 74) and fifth (high; n = 71) quintiles of Socio-Economic Index for Areas (SEIFA) were eligible to participate. Schools in each stratum were randomly ordered with probabilistic weighting according to enrolment number by the project coordinator and were approached consecutively within each stratum and invited to participate. After approaching 127 schools for participation, our target of 20 schools in low (n = 8), mid (n = 11), and high (n = 1) SEIFA areas was reached [19]. Schools were randomly allocated based on computer-generated blocks of four to one of three intervention groups (reducing sedentary behavior [SB], increasing physical activity [PA], or combined SB and PA [SB + PA]), or to a current practice control (C) condition, stratified by low or mid/high SEIFA areas [19]. Baseline data were collected from 7- to 10-year olds, their parents, and their teachers from February to June 2010 and mid-intervention (5–9 months) data were collected from November to December 2010. Specific details about the study, including the methodology and intervention, have been described previously [19].

**Figure 1:**
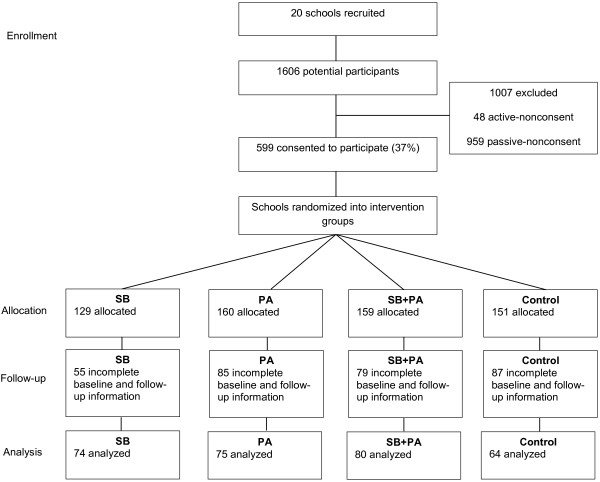
Flow of participants through the intervention.

All children in Grade 3 at the participating schools (N = 1606) and their parents were invited to participate in the Transform-Us! evaluation assessments in 2010. In total, 599 children (37%), 446 parents (28%), and 66 teachers (71%) completed baseline assessments and 567 children, 373 parents, and 49 teachers completed mid-intervention assessments. In Australia it is an ethics requirement for parents to provide active informed consent on behalf of themselves and their child; thus, it was not possible to obtain information regarding non-respondents. Figure 1 shows the flow of the participants through the intervention. Ethics approval was obtained from the Deakin University Human Research Ethics Committee (EC 141–2009) and the Victorian Department of Education and Early Childhood Development (2009_000344) and the Catholic Education Office (Project Number 1545).

### Intervention

The intervention was based on elements of the social cognitive theory (e.g., self-efficacy, observational learning, outcome expectancies), [20] behavioral choice theory (e.g., access, availability) [21], and the ecological systems theory (e.g., school policy) [22]. The aim of the SB arm was to reduce uninterrupted sedentary time during class in the school setting and overall sedentary time and discretionary screen time in the family setting. By mid-intervention, teachers were expected to have delivered nine out of 18 key learning messages (class lessons), one standing class lesson per day (~30 minutes), and a two-minute light-intensity activity break every 30 minutes within each two-hour teaching block. In addition, parents had received nine out of 18 newsletters supporting the key learning messages delivered to the children in the lessons [19].

The aim of the PA arm was to increase or maintain MVPA during recess and lunch breaks in the school setting and time spent outdoors in the family setting. While a specific focus to reduce sedentary time was not given in this arm, it is possible that sedentary time may have been reduced indirectly through the strategies to increase or maintain MVPA. Therefore, it is important to include this group in the analyses. By mid-intervention, teachers were expected to have delivered nine out of 18 lessons, promoted physical activity at recess and lunch breaks daily, made available the sporting equipment (e.g., balls, bats) supplied to each school at recess and lunch, and ensured that physical activity signage was displayed around the school. In addition, schools in the PA arm had novel asphalt line markings of their choice painted on their school playgrounds, and parents had received nine out of 18 newsletters supporting the key learning messages delivered to children in the lessons [19]. The combined SB + PA arm received a blended version of the PA (e.g., promotion of physical activity during recess and lunch breaks) and SB (e.g., standing lessons and activity breaks) interventions, with the same intervention dose [19]. The nine class lessons and newsletters encompassed both behaviors. After baseline data collection, teachers in the intervention schools received a half-day face-to-face professional development session to train them in the delivery of the intervention strategies. Children and schools in the control arm continued their usual practice.

### Outcome measure

#### Sedentary time

ActiGraph Model GT3X accelerometers were used to objectively assess children’s total sedentary time. Participants were asked to wear the accelerometers during waking hours on a belt positioned over the right hip for eight consecutive days (except when the accelerometer could get wet) at each of the measurement time points. Data were collected in 15-second epochs. Non-wear time was defined as a period of ≥20 minutes of consecutive zero counts. Previous research has suggested that longer bouts of consecutive zeros are biologically implausible in children [23], and this is the most commonly used non-wear definition in children [24]. A cut point of ≤100 counts per minute was used to define sedentary time [25]. Since the main objective of the intervention was to reduce uninterrupted sedentary time during class, average minutes of total sedentary time during class (classroom sedentary time) were calculated. Class time was defined by three periods including the start-of-school bell time until the start of recess, end of recess until the start of lunch, and end of lunch until the end of school bell time. Participants were required to have 50% of wear time within each class time period and have a minimum of two out of the three periods for computing overall class time [26]. Average minutes of total sedentary time on weekdays was calculated to determine whether a reduction in classroom sedentary time also resulted in a reduction of total weekday sedentary time or if children ‘compensated’ by increasing their sedentary time outside of class. Participants were required to have a minimum of eight hours of wear time per weekday for at least three weekdays to be included in the statistical analyses [27]. Therefore, while participants wore the accelerometer for eight consecutive days, only weekdays were analyzed in the present study. The distribution of valid weekdays in the sample at baseline showed that 8% of children had 0 weekdays of valid data, 6% had 1 weekday, 5% had 2 weekdays, 12% had 3 weekdays, 25% had 4 weekdays, and 44% had ≥5 weekdays. At mid-intervention, 4% of children had 0 weekdays of valid data, 11% had 1 weekday, 8% had 2 weekdays, 14% had 3 weekdays, 22% had 4 weekdays and 41% had ≥5 weekdays.

### Mediators

#### Intrapersonal Factor

##### Children’s enjoyment of standing in class

Children’s enjoyment of standing in class was measured with one item (5-point scale) at baseline and mid-intervention (how do you feel about standing up while doing your work during class). Higher scores reflected greater enjoyment of standing in class. One week test-retest reliability for the child enjoyment item was Kappa = 0.6 and per cent agreement = 86%.

#### Interpersonal factors

##### Parent outcome expectancies

Parents’ outcome expectancies towards their child standing in class were measured with six items (5-point scale) at baseline and mid-intervention (e.g., If my child spent more time standing during class time he/she would: concentrate more; be less productive in class; be tired when he/she gets home; be too tired to play outdoors after school; benefit his/her health; benefit his/her academic performance). One week test-retest reliability for the parent outcome expectancies items ranged from Kappa = 0.2 to 0.6 and per cent agreement = 66 to 87%. Responses from all six items were averaged to create an overall parental outcome expectancies score; higher scores reflected more positive outcome expectancies toward their child standing in class. The internal consistency for the parental outcome expectancies items in this study was Cronbach’s Alpha (α) = 0.8 at baseline and at mid-intervention.

##### Teacher outcome expectancies

Teachers’ outcome expectancies towards children standing in their class were measured with five items (5-point scale) at baseline and mid-intervention (e.g., Interrupted classroom lessons would: be too disruptive to the class; increase children’s ability to complete the task; negatively affect academic outcomes; result in children losing concentration; result in children being too disruptive). One week test-retest reliability for the teacher outcome expectancies items ranged from Kappa = 0.2 to 0.5 and per cent agreement = 33 to 78%. Responses from all five items were averaged to create an overall teacher outcome expectancies score; higher scores reflected more positive outcome expectancies towards children standing in class. The internal consistency for the teacher outcome expectancies items in this study was α = 0.8 at baseline and at mid-intervention.

#### Environment factor

##### Child perceived access to standing in the classroom environment

Children’s perceptions regarding access to opportunities to stand in the classroom environment [21] were measured with four items (3-point scale) at baseline and mid-intervention (e.g., My teacher: gets us to do lots of class activities standing up; gets us to move around a lot during class; encourages us to move around during class; makes sure we are not sitting down for a long time). One week test-retest reliability for the child access to standing items ranged from Kappa = 0.3 to 0.6 and per cent agreement = 64 to 79%. Responses from all four items were averaged to create an overall child access to standing score; higher scores reflected more positive perceptions of the classroom environment for access to standing in class. The internal consistency for the child access to standing items in this study was α = 0.6 at baseline and α = 0.7 at mid-intervention.

### Social-demographic characteristics

Socio-demographic characteristics measured at baseline by a parental questionnaire included child’s sex, country of birth (Australia, other), and socioeconomic position (SEP). The self-reported highest level of maternal education was used as a proxy-measure of SEP and classified as low (schooling < 12 years), medium (schooling = 12 years), and high (tertiary education). Child’s age was also assessed by a parental questionnaire at both baseline and mid-intervention.

### Statistical analysis

Analyses were conducted using SAS version 9.2 [SAS Institute Inc., Cary, NC] in 2012. The MIXED and SURVEYFREQ procedures were used to account for the hierarchical and clustered nature of the data. Descriptive statistics were calculated. The assumption of normality for regression models of classroom sedentary time, total weekday sedentary time, and mediating variables were assessed by examining residuals. One-way ANOVAs and chi-square tests were conducted to examine whether baseline differences existed between groups. Repeated measures one-way ANOVAs were conducted to examine whether accelerometer wear time and valid weekdays differed between baseline and mid-intervention.

**Figure 2:**
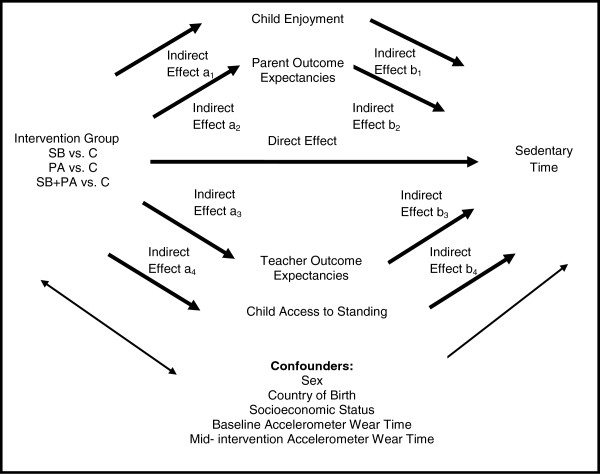
**The direct and indirect mid-intervention effects on sedentary behavior, when considering child enjoyment, parent and teacher outcome expectancies, and child access to standing in the classroom environment as mediator variables**[30]**.**

To address the main study objective, the total, direct, and indirect mid-intervention effects on sedentary time were estimated. The total effect represents the mid-intervention effect on sedentary time, without adjusting for mediators. The direct effect represents the mid-intervention effect on sedentary time, after adjusting for mediators (Figure 2). The indirect effect represents the mid-intervention effect on sedentary time that occurred through the mediators (Figure 2). Separate analyses were performed for classroom sedentary time and total weekday sedentary time. Sample size calculations for the mediation analyses were based on a 2007 simulation study by Fritz and MacKinnon [28]. To detect a moderate mediated effect size (standardized regression coefficients a and b of ~0.39) [29] with 0.8 power, using a significance level of 0.05, with a two-tailed test, and percentile bootstrap methods, ~78 observations are needed without accounting for school cluster effects [28].

The total and direct effects were estimated using multilevel linear regression models. The indirect effects were estimated using a bootstrap sampling procedure with 1000 resamples, that took into account multiple mediators [30,31]. This procedure calculated point estimates and bootstrap percentile 95% confidence intervals based on the product of regression coefficients estimated in multilevel linear regression models (indirect effects a_1_*b_1_ and a_2_*b_2_ and a_3_*b_3_ and a_4_*b_4_ in Figure 2) for each resample [30]. There was evidence of mediation if zero was not included in the confidence interval [30]. Multiple mediator models were used to estimate the indirect effect of one mediator variable while controlling for the effects of other mediators. Findings from simulation research suggest that this bootstrap sample procedure is one of the most powerful and accurate methods for testing mediation [30]. Sex, country of birth, SEP, baseline accelerometer wear time, mid-intervention accelerometer wear time, and baseline mediator variables were considered as potential confounders in all regression analyses. Child’s age was not included because of the homogenous age-group targeted for the intervention.

Along with the analyses performed to address the total, direct, and indirect effects, multilevel linear regression models were used to examine the mid-intervention effect on the mediator variables (path a_1_, a_2_, a_3_, a_4_ in Figure 2) and the effect of the mediator variables on classroom sedentary time and total weekday sedentary time (path b_1_, b_2_, b_3_, b_4_ in Figure 2). These analyses also controlled for the confounders used in the main analyses.

The intention to treat principle was used, where baseline data were imputed for missing mid-intervention sedentary behavior or mediator data among 110 participants and mid-intervention data were imputed for missing baseline sedentary behavior or mediator data among 35 participants. We excluded 306 children with incomplete baseline and mid-intervention sedentary behavior or mediator data. In total, 293 children were included in the final analyses (Figure 1). There were no significant age, sex, or group differences between the included and excluded participants (*P* > 0.05). There were also no significant differences at mid-intervention for any of the variables of interest between participants missing and not missing baseline data (*P* > 0.05), with the exception of SEP and country of birth. Those with missing baseline data had a lower percentage (17% versus 45%) of parents with high SEP and a higher percentage of Australian-born parents (64% versus 24%). Finally, there were no significant differences at baseline for any of the variables of interest between participants missing and not missing mid-intervention data (*P* > 0.05), with the exception of classroom accelerometer wear time. The baseline classroom accelerometer wear time for participants with missing mid-intervention data (290.8 min/day) was significantly higher than for participants not missing mid-intervention data (280.7 min/day).

## Results

### Descriptive analyses

At baseline, approximately 56% of the overall sample was female and the average age was 8.0 (1.3 SD) years (Table 1). On average, participants in the overall sample had 4.2 (1.5 SD) valid weekdays at baseline and 4.1 (1.6 SD) valid weekdays at mid-intervention. On average, participants in the overall sample had 284.5 (28.3 SD) min/day at baseline and 289.8 (20.0) min/day at mid-intervention of classroom accelerometer wear time. On average, participants in the overall sample had 532.5 (109.8 SD) min/day at baseline and 533.3 (113.7) min/day at mid-intervention of total weekday accelerometer wear time. There were no significant group differences at baseline in the children’s age, sex, SEP, parent’s country of birth, classroom sedentary time, total weekday sedentary time, classroom accelerometer wear time, total weekday accelerometer wear time, valid weekdays, or any of the mediator variables (*P* > 0.05). Likewise, there were no significant group differences in classroom accelerometer wear time, total weekday accelerometer wear time, or valid weekdays at mid-intervention (*P* > 0.05). However, a significant difference existed in classroom accelerometer wear time of 5 min/day between baseline and mid-intervention (*P* = 0.002) but not for total weekday accelerometer wear time or number of valid weekdays.

**Table 1:** Baseline participant characteristics, sedentary time, and mediator variables by intervention group

	**SB**	**PA**	**SB + PA**	**Control**	**Total**
**Variable**	**(n = 74)**	**(n = 75)**	**(n = 80)**	**(n = 64)**	**(N = 293)**
Child age (years)	7.9 (1.4)	7.8 (1.4)	8.0 (1.4)	8.1 (0.4)	8.0 (1.3)
Child sex (%)					
Boys	36.5	47.4	51.2	40.6	44.2
Girls	63.5	52.6	48.8	59.4	55.8
Parental education (%)					
Low	13.5	14.5	17.5	12.5	14.6
Medium	37.8	38.1	45.0	53.1	43.2
High	48.7	47.4	37.5	34.4	42.2
Country of birth (%)					
Australia	70.3	67.1	61.2	75.0	66.9
Other	29.7	32.9	38.8	25.0	33.1
Classroom sedentary time (% of wear time)					
Baseline	58.9 (5.9)	60.4 (6.4)	62.1 (7.0)	60.8 (5.8)	60.6 (6.4)
Mid-intervention	60.1 (6.6)	60.1 (6.2)	60.2 (6.8)	62.7 (4.8)	60.7 (6.3)
Weekday sedentary time (min/day)					
Baseline	62.9 (7.5)	64.4 (8.3)	63.4 (6.7)	64.6 (6.1)	63.8 (7.2)
Mid-intervention	61.5 (7.8)	64.4 (6.9)	62.5 (6.6)	63.0 (6.0)	62.8 (6.9)
Child enjoyment of standing in class^a^					
Baseline	2.2 (1.4)	2.4 (1.3)	2.5 (1.3)	2.2 (1.4)	2.3 (1.3)
Mid-intervention	3.3 (1.3)	2.9 (1.3)	2.9 (1.4)	2.4 (1.4)	2.9 (1.4)
Parent outcome expectancies of child standing in class^a^					
Baseline	3.0 (0.6)	2.9 (0.6)	3.0 (0.6)	2.8 (0.6)	2.9 (0.6)
Mid-intervention	3.2 (0.6)	3.0 (0.6)	3.1 (0.6)	2.9 (0.6)	3.1 (06)
Teacher outcome expectancies of children standing in class^a^					
Baseline	2.2 (0.4)	2.4 (0.4)	2.5 (0.7)	2.5 (0.7)	2.4 (0.6)
Mid-intervention	2.1 (0.5)	2.3 (0.7)	2.1 (0.6)	2.3 (0.5)	2.2 (0.6)
Child perceived access to standing opportunities in the classroom environment^b^					
Baseline	1.6 (0.4)	1.6 (0.5)	1.6 (0.4)	1.6 (0.4)	1.6 (0.4)
Mid-intervention	1.9 (0.4)	1.8 (0.5)	1.8 (0.4)	1.5 (0.3)	1.8 (0.4)

PA = physical activity; SB = sedentary behavior.

Data presented as mean (standard deviation) for continuous variables and % for categorical variables.

There were no significant baseline group differences for any of the variables.

^a^Range of values was 1–5; ^b^Range of values was 1–3.

### Mid-intervention effects on sedentary time

#### Total effects

The mid-intervention effects on sedentary time, without adjusting for mediators, but after adjusting for confounders (sex, country of birth, SES, baseline accelerometer wear time, mid-intervention accelerometer wear time, baseline sedentary time, and baseline mediator variables), are shown in Table 2. For total weekday sedentary time, a significant mid-intervention total effect was observed for the SB + PA group. More specifically, the SB + PA group had 13.3 min/day lower total weekday sedentary time at mid-intervention compared to the control group. No mid-intervention total effect on total weekday sedentary time was observed for the SB or PA groups. For classroom sedentary time, no mid-intervention total effects were observed for any of the intervention groups compared with the control group.

**Table 2:** **Total**^**a**^**and direct**^**b**^**mid-intervention effects on sedentary time (min/day)**

	**Total effect**		**Direct effect**	
**Intervention group**	**Classroom**	**Total weekday**	**Classroom**	**Total weekday**
	**β (95% CI)**	**β (95% CI)**	**β (95% CI)**	**β (95% CI)**
Control	Referent	Referent	Referent	Referent
SB	−1.12 (−7.23, 4.99)	−9.0 (−20.92, 2.93)	0.17 (−6.14, 6.48)	−6.90 (−19.50, 5.69)
PA	3.78 (−2.06, 9.62)	−8.91 (−20.10, 2.27)	4.45 (−1.46, 10.36)	−7.69 (−19.50, 5.69)
SB + PA	1.87 (−3.85, 7.60)	−13.28 (−24.37, 2.20)*	3.22 (−2.74, 9.18)	−11.36 (−23.16, 0.45)

PA = physical activity; SB = sedentary behavior.

^a^The total effect models were adjusted for sex, country of birth, SES, baseline accelerometer wear time, mid-intervention accelerometer wear time, baseline sedentary time, and baseline mediator variables.

^b^The direct effect models were adjusted for all the variables in the total effect models and mid-intervention mediator variables.

* *P* < 0.05.

#### Direct effects

The mid-intervention effects on sedentary time, after adjusting for mediators and confounders, are also shown in Table 2. For classroom sedentary time and total weekday sedentary time, no mid-intervention direct effects were observed for any of the intervention groups compared with the control group.

#### Indirect effects

The mid-intervention effects on sedentary time that occurred through the mediators after adjusting for confounders are shown in Table 3. Child enjoyment, parent and teacher outcome expectancies and child access to standing were not significant mediators of classroom sedentary time or total weekday sedentary time at mid-intervention (Table 3).

**Table 3:** Indirect^a^ mid-intervention effects on sedentary time (min/day)

	**Specific indirect effects**				**Total indirect effect**
	**Child enjoyment**	**Parent outcome expectancies**	**Teacher outcome expectancies**	**Child access to standing**	
**Intervention group**	**P. Est. (95% CI)**	**P. Est. (95% CI)**	**P. Est. (95% CI)**	**P. Est. (95% CI)**	**P. Est. (95% CI)**
Classroom					
Control	Referent	Referent	Referent	Referent	Referent
SB	−0.29 (−1.39, 0.67)	−0.19 (−0.87, 0.48)	−0.07 (−0.58, 0.21)	−0.57 (−1.94, 0.80)	−1.12 (−2.91, 0.69)
PA	−0.08 (−0.57, 0.24)	−0.0004 (−0.32, 0.32)	0.09 (−0.14, 0.41)	−0.45 (−1.58, 0.62)	−0.44 (−1.65, 0.80)
SB + PA	−0.14 (−0.82, 0.36)	−0.21 (−0.92, 0.55)	−0.20 (−0.67, 0.25)	−0.60 (−2.06, 0.83)	−1.15 (−2.86, 0.46)
Total weekday					
Control	Referent	Referent	Referent	Referent	Referent
SB	−0.58 (−2.91, 1.56)	−0.15 (−1.52, 1.26)	0.14 (−0.26, 1.16)	−1.55 (−4.96, 1.45)	−2.14 (−6.27, 1.81)
PA	−0.16 (−1.17, 0.54)	0.02 (−0.51, 0.63)	0.0002 (−0.50, 0.54)	−1.20 (−3.91, 1.18)	−1.33 (−4.16, 1.16)
SB + PA	−0.29 (−1.65, 0.80)	−0.16 (−1.54, 1.50)	0.01 (−0.98, 1.09)	−1.61 (−5.13, 1.66)	−2.06 (−5.87, 1.40)

P. Est. = point estimate; 95% CI = Bootstrap percentile 95% confidence interval; PA = physical activity; SB = sedentary behavior.

^a^All models are adjusted for sex, country of birth, SES, baseline accelerometer wear time, mid-intervention accelerometer wear time, baseline sedentary behavior, and baseline mediator variables.

*P < 0.05.

### Mid-intervention effects on the mediating variables (Paths a_1_, a_2_, a_3_, a_4_)

Additional analyses revealed that after adjusting for confounders there was a significant mid-intervention effect on child enjoyment for the SB group (Table 4). Child enjoyment at mid-intervention was 0.9 units (5-point scale) higher in the SB group compared to the control group. There was also a significant mid-intervention effect on child access to standing for all intervention groups. Child access to standing scores at mid-intervention were 0.4 units (3-point scale) higher (more positive perception) in the SB group, 0.3 units higher in the PA group, and 0.4 units higher in the SB + PA group compared to the control group.

**Table 4:** Mid-intervention effects on the hypothesized mediating variables^a^

	**Child enjoyment**	**Parent outcome expectancies**	**Teacher outcome expectancies**	**Child access to standing**
**Intervention group**	**β (95% CI)**	**β (95% CI)**	**β (95% CI)**	**β (95% CI)**
Control	Referent	Referent	Referent	Referent
SB	0.88 (0.36, 1.41)*	0.15 (−0.01, 0.30)	−0.01 (−0.47, 0.45)	0.35 (0.14, 0.55)*
PA	0.33 (−0.18, 0.83)	0.001 (−0.15, 0.15)	0.05 (−0.35, 0.45)	0.27 (0.09, 0.46)*
SB + PA	0.47 (−0.03, 0.97)	0.14 (−0.009, 0.30)	−0.12 (−0.53, 0.28)	0.37 (0.18, 0.55)*

PA = physical activity; SB = sedentary behavior.

^a^All models are adjusted for sex, country of birth, SES, and baseline mediator variables.

*P < 0.05.

### The effects of the mediating variables on sedentary behavior (Paths b_1_, b_2_, b_3_, b_4_)

Additional analyses revealed that after adjusting for confounders child enjoyment, parent and teacher outcomes expectancies, and child access to standing did not have a significant effect on classroom sedentary time or total weekday sedentary time at mid-intervention (Table 5).

**Table 5:** Effects of the hypothesized mediating variables on sedentary time (min/day)^a^

	**Classroom**	**Total weekday**
**Mediating variables**	**β (95% CI)**	**β (95% CI)**
Child enjoyment	−0.35 (−1.59, 0.89)	−0.73 (−3.16, 1.17)
Parent outcome expectancies	−0.82 (−4.41, 2.77)	−0.82 (−7.84, 6.20)
Teacher outcome expectancies	1.47 (−2.63, 5.57)	−0.21 (−8.31, 7.88)
Child access to standing	−2.09 (−6.35, 2.16)	−3.78 (−12.24, 4.68)

^a^All models are adjusted for sex, country of birth, SES, baseline accelerometer wear time, mid-intervention accelerometer wear time, baseline sedentary time, baseline mediator variables, and the intervention group.

*P < 0.05.

## Discussion

This study examined whether child enjoyment, parent and teacher outcome expectancies, and the perceived access to standing opportunities in the classroom environment mediated the mid-intervention effects on children’s classroom sedentary time or total weekday sedentary time in the Transform-Us! cluster-randomized controlled trial. While a significant mid-intervention effect was found for total weekday sedentary time in the SB + PA group, no significant mediating effects were found. A significant mid-intervention effect was also observed on child-reported enjoyment of standing during class time in the SB group and perceived access to standing opportunities in the classroom environment in all intervention groups; however, these hypothesized mediator variables did not have a significant effect on sedentary time at mid-intervention.

While no previous study has examined the mediating mechanisms of interventions aiming to reduce children’s total sedentary time, three studies have examined potential mediators of screen time interventions [17]. Similar to the present study, no significant mediating effects were observed in any of the studies [17]. More specifically, none of the examined mediators (TV allowance use, additional TV allowance requests, proportion of newsletters read by parents, the number of incentives given, and the number of days with no screen time during the ‘TV Turnoff’ period) were found to mediate the intervention effect on children’s screen time in the SMART randomized controlled trial [32]. Likewise, meanings of physical activity (i.e., personal, social, functional, fantasy) and motivation for physical activity (i.e., external regulation, introjected, identified, intrinsic, relative autonomy index) did not significantly mediate the intervention effect observed on girls’ screen time in the Get Moving! Intervention [33]. However, the mediating effect of intrinsic motivation approached significance. Additionally, no mediating effects were found for youth attitudes, subjective norms, perceived behavioral control, or habit strength in the DOiT cluster-randomized controlled trial, which also targeted youth’s screen time along with other energy balance related behaviors [34].

One potential reason for the lack of mediating effects in the previous literature and the present study may be the measurement quality of the self-reported mediator variables [17]. The reliability of some of the individual items was either not available or in the case of the present study was low to moderate (Kappa = 0.2-0.5; per cent agreement = 33-78%). However, these measures were analysed as score items with most showing moderate to high internal consistency (α = 0.6-0.8 in the present study) [33,34]. Additionally, the sensitivity of the mediator variables to capture change is unknown [17]. In addition, the lack of mediating effects could be explained by the failure to capture the relevant mechanisms that explain the intervention effects on sedentary time. For example, the investigators of the Get Moving! Intervention included mediators from the physical activity literature that were not specific to screen time [33]. Although the mediators in the present study were specific to sedentary time, they focused on class time. Consequently, mediators that focused on time outside of class (e.g., parental outcome expectancies towards standing while completing homework) may have been more relevant, given that an intervention effect was observed for total weekday sedentary time but not classroom sedentary time. Finally, given this study examined mid-intervention effects, it is possible that mediating effects were not observed because of a time lag between the change in mediator variables and the impact of this change on sedentary time [35]. For example, in order to effect change in sitting time, children may need to embrace opportunities (access) to stand and move during class lessons as enjoyable activities. Thus, the mid-intervention effects observed on child enjoyment and perceived access to standing opportunities in the classroom environment suggest the intervention is on the right track and changes in sedentary time may be observed later on in the intervention.

This study makes an important contribution to the literature by being the first to examine the mediating effects on children’s objectively assessed total sedentary time; however, a dearth of information still exists on the optimal targets and strategies for effectively reducing sedentary behavior. Therefore, researchers conducting sedentary behavior interventions in the future should conduct mediation analyses. This research should include reliable measures of mediators that are sensitive to change over time [17,36]. This will enable more effective interventions to be developed in the future that may translate into improved health outcomes among children.

Strengths of this study include the objective measures of total sedentary time and the contemporary mediation analysis. While the accelerometer may have classified some time spent sitting and standing, previous work in this age group has shown small mean bias between sitting measured by the activPAL monitor and sedentary time measured by the ActiGraph accelerometer with a cut-point of ≤100 counts per minute, especially during school hours [25]. In addition, defining 20 minutes of consecutive zeros may have underestimated total sedentary time in this sample, though future research is needed to identify an appropriate criterion for defining non-wear in children. Furthermore, the child and parent response rates were less than optimal. The requirement for active consent in Australia may explain this lower response rate. Recent research regarding school-based questionnaires reported that changing from passive to active consent in Australia resulted in response rate reductions from 90-97% to 37-40% [37]; the latter is comparable with the current study. In addition, approximately half of the sample was excluded from the analyses due to incomplete data at both assessments; while, there were no significant age, gender, or group differences between included and excluded participants, the study may have been underpowered to detect multiple independent mediated effects. Also, since a number of participants had baseline or mid-intervention data imputed, the overall changes in sedentary behavior or the mediating variables may have been attenuated. Further work is required to determine whether this intervention program is applicable to adolescents, rural populations, and other countries.

## Conclusions

While the intervention had beneficial effects on child enjoyment, child perceived access to standing opportunities in the classroom environment, and total weekday sedentary time, no significant mediating effects were found. Due to limitations in the existing literature and the overall dearth of information, optimal targets and strategies for effectively reducing sedentary behavior remain poorly understood. Future intervention research is needed that explores mediated effects. More work is also needed on the development of reliable mediator measures that are sensitive to change overtime.

## Competing interests

The authors declare that they have no competing interests.

## Authors’ contributions

JS took the lead in designing the study and secured the funding. LA managed the data collection. VC performed the statistical analyses. All authors interpreted the data. VC wrote the manuscript. All authors critically reviewed and revised the manuscript for important intellectual content. All authors read and approved the final version of the manuscript.
